# Genomic Variations in Pancreatic Cancer and Potential Opportunities for Development of New Approaches for Diagnosis and Treatment

**DOI:** 10.3390/ijms18061201

**Published:** 2017-06-05

**Authors:** Shuangshuang Lu, Tasqeen Ahmed, Pan Du, Yaohe Wang

**Affiliations:** 1 National Center for International Research in Cell and Gene Therapy, Sino-British Research Centre for Molecular Oncology, School of Basic Medical Sciences, Academy of Medical Sciences, Zhengzhou University, Zhengzhou 450052, China; lushuangshuang@zzu.edu.cn (S.L.); dupan1012@163.com (P.D.); 2 Centre for Molecular Oncology, Barts Cancer Institute, Queen Mary University of London, London EC1M 6BQ, UK; t.ahmed48@outlook.com

**Keywords:** pancreatic cancer, genomic variations, driver mutations, diagnosis, therapy

## Abstract

Human pancreatic cancer has a very poor prognosis with an overall five-year survival rate of less than 5% and an average median survival time of six months. This is largely due to metastatic disease, which is already present in the majority of patients when diagnosed. Although our understanding of the molecular events underlying multi-step carcinogenesis in pancreatic cancer has steadily increased, translation into more effective therapeutic approaches has been inefficient in recent decades. Therefore, it is imperative that novel and targeted approaches are designed to facilitate the early detection and treatment of pancreatic cancer. Presently, there are numerous ongoing studies investigating the types of genomic variations in pancreatic cancer and their impact on tumor initiation and growth, as well as prognosis. This has led to the development of therapeutics to target these genetic variations for clinical benefit. Thus far, there have been minimal clinical successes directly targeting these genomic alterations; however research is ongoing to ultimately discover an innovative approach to tackle this devastating disease. This review will discuss the genomic variations in pancreatic cancer, and the resulting potential diagnostic and therapeutic implications.

## 1. Introduction

Over the last few decades, pancreatic cancer has consistently remained one of the most lethal and challenging cancers to diagnose and treat [1]. Although significant advances have been made in the domain of cancer treatment to enhance survival rates for other solid tumors including breast, colorectal, prostate and renal, mortality rates for pancreatic cancer patients remain extraordinarily high. Additionally, pancreatic cancer statistics have proven to be disappointing with 90,100 new pancreatic cancer cases and 79,400 deaths in China in 2015 [2], as well as 53,070 new pancreatic cancer cases and 41,780 estimated deaths in United States in 2016 [3]. Moreover, prognoses for patients remain devastatingly poor. In particular, patients presenting with locally advanced cancer have a median survival time of 8–12 months, whereas those presenting with distant metastases have an exceedingly worse prognosis with a median survival time of 3–6 months. As well as this, five-year survival rates are still less than 5% despite 50 years of research and therapeutic development [4,5]. Extensive research has categorized pancreatic cancer into two main subtypes: exocrine and neuroendocrine types. Pancreatic ductal adenocarcinoma (PDAC) belongs to the exocrine group and is the most common type, dominating about 85% of all pancreatic cancer cases [1].

At present, the majority of patients present with locally advanced or metastatic pancreatic cancer. Delayed diagnosis is a result of a multitude of factors, including non-specific symptoms such as anorexia, jaundice and gastric outlet obstructions. Additionally, there are no effective screening methods for low-risk cohorts, which hinders early detection, as hereditary factors only contribute to 5–10% of pancreatic cancer cases. Thus, patients present at a late stage in their disease, which reduces treatment options and complicates manageability of the disease [5,6]. Currently, the only potential curative option is surgery, however, only a mere 15–20% of patients are able to undergo pancreatectomy when diagnosed. Coupled with the fact that over 80% of patients relapse after resection, surgery does not constitute a promising treatment option [7]. Moreover, there are limited chemotherapy agents that have clinical benefits in pancreatic cancer and response rates vary significantly. Presently, gemcitabine-based treatments remain the first-line therapy for pancreatic cancer patients with gemcitabine combined alongside erlotinib being the standard treatment regimen for patients with advanced disease. However, clinical outcomes are still marginal. Even within the metastatic setting, chemotherapy regimens have rendered only modest improvements in survival rates. For example, gemcitabine combined with nab-paclitaxel demonstrated minor improvements in overall survival with a median survival of one year for patients with advanced disease [4]. Additionally, FOLFIRINOX (folinic acid, 5-fluorouracil, irinotecan, and oxaliplatin) has recently also been implicated in metastatic disease with marginal clinical success [8]. A potential explanation for late diagnosis is that molecular carcinogenesis of PDAC occurs over decades, through stepwise progression from a preinvasive stage, pancreatic intraepithelial neoplasia (PanIN), to invasive pancreatic cancer. This evolution correlates with accumulation of genetic aberrations and other molecular irregularities [9]. Pancreatic cancer is characterized by four major driver genes: Kirsten rat sarcoma viral oncogene homolog (*KRAS*), Cyclin-dependent kinase Inhibitor 2A (*CDKN2A*), Mothers against decapentaplegic homolog 4 (*SMAD4*) and Tumor protein p53 *(TP53*). *KRAS* is of particular importance as the activating point mutation in the *KRAS* oncogene found in 90% of pancreatic cancer cases and it comprises one of the initial genetic mutations in non-cancerous precursor lesions [10]. Additionally, the tumor suppressor gene *CDKN2A*, which regulates G1 to S phase in the cell cycle, undergoes a mutational inactivation. Finally, *SMAD4* and *TP53* are primarily found in higher-grade lesions [5].

The dismal survival rates for pancreatic cancer, as well as the disappointing response rates and high levels of resistance to standard treatments has reinforced the urgent and crucial need for novel treatments for pancreatic cancer patients. This has forged an avenue for the study of genomic variations on pancreatic cancer, which have been demonstrated to play a role in initiation, development and invasion of the disease. Thus, these genetic alterations may provide insight into targets for diagnosis and innovative and effective treatments for patients. In line with this, next-generation DNA sequencing technologies have revolutionized the study of genomic variations in cancer and are pioneering the developmental direction of precision medicine. This technology provides the advantage of increasing the speed of gaining results and reducing costs. Fully analyzing the genomic landscapes of tumors is highly beneficial as this can pave the way for improved diagnosis and prognosis. Moreover, there are many institutions dedicated to characterizing the genomic and transcriptomic landscapes of cancer, including The Cancer Genome Atlas and the International Cancer Genome Consortium, which should hasten progress in this field.

This review will discuss the genomic variations in pancreatic cancer, and the diagnostic as well as therapeutic implications of this will be provided.

## 2. Types of Genomic Variations

Through whole-genome and deep-exome sequencing, genomic multiplicities of pancreatic cancer, such as copy number alterations, point mutations and indels, chromosomal aberrations and epigenetic changes have been identified. Interestingly, pancreatic cancer has fewer mutations in comparison to other cancer types [11]. One potential explanation is that the initiating cells of pancreatic cancer undergo fewer divisions. In this part, we discuss a variety of genomic variations within pancreatic cancer.

### 2.1. Chromosomal Aberrations

Chromosomal aberrations in pancreatic cancer include loss, gain and structural rearrangements. It has been demonstrated that losses of chromosome 18 (78%), 17 (56%), 6 (44%), 21 (42%), 22 (42%), Y (36%), 4 (33%) and gain of chromosome 20 (28%) are very common in pancreatic adenocarcinomas [12]. Furthermore, there are three types of structural rearrangements: translocations, gene fusions and inversions. However, structural rearrangements are relatively uncommon in the pancreatic cancer genome [13,14]. Somatic structural variants were identified with the qSV package in 100 pancreatic cancer patients, totally, 11,868 somatic structural variants were identified and every individual has about 119 variants on average [14].

### 2.2. Copy Number Variations

Copy number variations (CNV) include: amplifications, deletions and loss of heterozygous (LOH) and are prevalent in the whole genome of pancreatic cancer [15,16,17]. High levels of amplifications at 7q21.3–q22.1 and 19q13.2 and homozygous deletions at 1p33–p32.3, 1p22.1, 1q22, 3q27.2, 6p22.3, 6p21.31, 12q13.2, 17p13.2, 17q21.31 and 22q13.1 have been reported from 93 pancreatic carcinoma patients in China using Array-Based Comparative Genomic Hybridization [18]. Furthermore, analysis of genomic sequences of 456 patients in the Australian Pancreatic Cancer Genome Initiative (APGI), revealed 50 regions of amplifications and 73 regions of deletions [13]. The alternated regions contained oncogenes, such as *KRAS*, *MET*, *NOTCH*, and *GATA6* and tumor suppressor genes, including *CDKN2A*, *SMAD4*, and *TP53* [13,16,19]. The number of CNV is relative to the postoperative survival, thus usually patients with more CNV have shorter survival rates [15,20].

### 2.3. Point Mutations and Indels

Point mutations and indels are common in the whole genome of pancreatic cancer [14,17,21,22,23,24], with many oncogenes and tumor suppressor genes existing in their mutated forms. *KRAS*, *TP53*, *CDKN2A* and *SMAD4* are most prevalent mutated genes in pancreatic cancer [14,21,25,26,27,28,29,30]. KRAS is activated by point mutations in 95% of invasive ductal adenocarcinomas. The KRAS point mutations present early in pancreatic neoplasia, and almost exclusively target three codons (12, 13 and 61), which are relatively easy to identify. *KRAS* mutations can be used as diagnostic markers to detect early curable pancreatic neoplasia [25,29]. Additionally, the *TP53* tumor suppressor gene on chromosome 17p has lost its function in 75% of pancreatic cancers [27]. Moreover, another tumor suppressor gene on chromosome 9p*P16*, *CDKN2A* is inactivated in about 95% of pancreatic cancers [26,28,31]. The fourth major alteration of tumor suppressor gene on chromosome 18q is *SMAD4* (previously called *DPC4*) [30].

### 2.4. Epigenetic Changes

The major epigenetic changes in pancreatic cancers include DNA methylation and microRNA profiles [16]. Both of these can potentially alter gene expressions in cancer cells. In particular, many important genes involved in the progression of pancreatic cancer have been hypermethylated, such as *RARB* [32], *APC* [33], *TSLC/IGSF4* [34], *SOCS-1* [35], *CCND2* [36], *RASSF1A* [37], *WWOX* [38], *RUNX3* [39], *CDH13* [40], *HHIP* [41], *SLC5A8* [42] and *P16/CDKN2A* [32,43,44]. Hypermethylated promoters of tumor suppressor genes result in their inactivation and facilitate tumorigenesis.

In addition, 18–24 bases long microRNAs (miRNAs) can regulate the stability and translational efficiency of the targeting mRNAs and are involved in cell proliferation, cell death and tumorigenesis [45]. The miRNA profiles are unique in various cancer types [46]. A group of 112 miRNAs, which vary among pancreatic cancer tissues, normal and benign tissues, including in pancreatitis have been identified [47]. miR-217 and miR-196a expression profiles can be used in differentiating pancreatic cancer tissue from normal pancreas tissues and chronic pancreatitis [48]. Furthermore, miR-21 was found significantly up-regulated in 20 pancreatic carcinomas and six cancer cell lines compared with the coupled benign tissues and normal pancreas [49]. miR-196a-2 was identified to be a predictor of survival of pancreatic tumor patients [50]. Overall, abnormalities in microRNA expression within endocrine and acinar pancreatic tumors are associated with distinctive pathologic features and clinical behaviors.

## 3. Contribution of Genomic Instability to Pancreatic Carcinogenesis

Through decades of research, scientists have demonstrated that tumors possess ten unifying traits: sustained proliferation signaling; growth suppressor evasion; cell death resistance; enabling of replicative immortality; induction of angiogenesis; activation of invasion and metastasis; evasion of immune destruction; deregulated cellular energetics; tumor promoting inflammation and genome instability and mutation [51].

**Figure 1:**
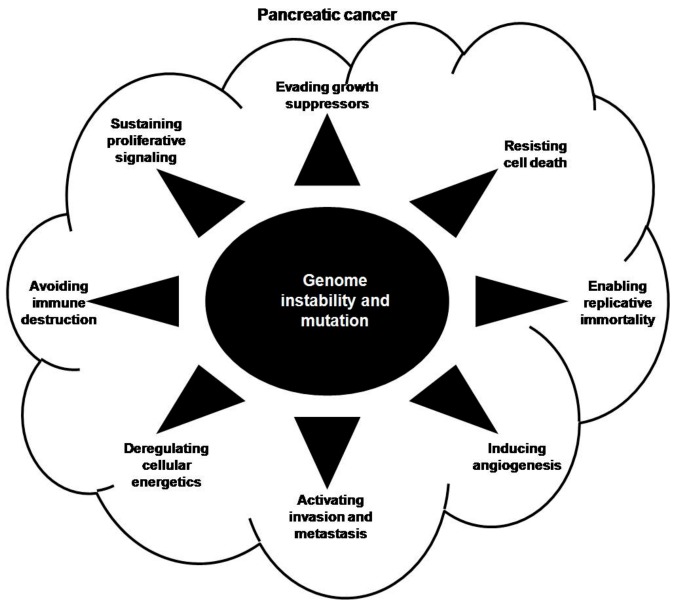
Contributions of genomic variations to pancreatic carcinogenesis

Genomic instability and mutation is an enabling characteristic because it can induce the acquisition of other hallmarks of cancer (Figure 1). Several different pathways contribute to one trait acquisition, resulting in various pathways becoming activated to facilitate carcinogenesis when one pathway is blocked. Mutations of some genes participate in more than one trait acquisition. In this part, we discuss the contribution of genomic variations to pancreatic carcinogenesis (summarized in Table 1).

### 3.1. Sustained Proliferation

In normal tissues, the production and release of growth-promoting signals are carefully controlled to ensure homeostasis of cell number and maintain normal tissue architecture and function. In pancreatic cancer cells, control of these signals is lost. Somatic mutations such as *phosphoinositide3-kinase (PI3K)*-*AKT/PKB* [52,53] and *KRAS* [54] constitutively activate proliferation signaling. Mutations in *PTEN* [53] disrupt the negative feedback of proliferation signaling. All of these genetic mutations contribute to sustained proliferation of pancreatic cancer cells.

### 3.2. Evading Growth Suppression

In normal cells, growth suppression signals inhibit excessive growth; on the contrary, cancer cells have the ability to evade growth suppression. Mutations in tumor suppressor genes such as *TP53* and *RB* [55,56,57] that participate in the inhibition of cell cycle progression result in cells losing control of growth suppression. As well as this, decreased expression of *NF2* [58,59] and *LKB1* [60,61,62] genes in pancreatic tumor and cancer cell lines also contribute to evading growth suppression. In particular, the *NF2* gene encodes Merlin, a member of the band 4.1 family of cytoskeleton-associated proteins, functions as a tumor suppressor and is a critical mediator of contact dependent inhibition of growth through signals from the extracellular matrix and *FOXM1/WNT/β-CATENIN* pathway in pancreatic cancer [63]. It has been elucidated that *LKB1* (*liver kinase B1*) can regulate cell proliferation and polarity and its expression influences PDAC patients overall survival.

### 3.3. Resisting Cell Death

Cancer cells can avoid programmed cell death by abnormal expression of oncogenes such as *CCND1* [64], *BCL-2* [65] and inactivation of tumor suppressor genes such as *TP53* and *BRCA2* [66].

Previous research demonstrated that autophagy can protect cancer cells from death induced by cetuximab [67]. When depriving nutrients or growth factors that govern nutrient uptake, cell autophagy, a regulated catabolic process, will happen. Autophagy is a double-edged sword in cancer as it participates both in cytoprotection and in cell death [68,69]. The presence of hypoxia in pancreatic cancer induces autophagy in cancer cells [70]. *BECLIN1*, a critical autophagic gene, mediates autophagy of pancreatic cancer cells. *miRNA-216a* targets *BECLIN1* by directly interacting with its 3′-untranslated region to inhibit it [71]. In pancreatic cancer, miRNA-216a is significantly downregulated and cells can antagonize death through *BECLIN1*-mediated autophagy [71,72].

### 3.4. Enabling Replicative Immortality

Replicative immortality depends on the stability of telomeres as it maintains the integrity of chromosomes and inhibits DNA degradation. In normal cells, telomeres are consumed and shortened in each DNA replication process. Till the threshold of the telomere length, cells go to senescence. Telomerase, composed of template RNA (transcript from *hTR* gene), telomere related protein 1 (*TP1*) and telomerase reverse transcriptase (*hTERT*), is responsible for maintaining the length of telomeres. In normal mature somatic cells, the activity of telomerase cannot be detected. *Protocadherin 10* (*PCDH10*), a tumor suppressor, was found to negatively regulate telomerase activity through interaction with *hTERT* [73]. Whereas, the promoter of *PCDH10* was hypermethylated and its expression was decreased in pancreatic cancer [74] thus elevating telomerase activity to enable replicative immortality of cancer cells [75,76].

### 3.5. Inducing Angiogenesis

Erythroblastosis virus E26 oncogene homolog 1 (*ETS-1*), a transcription factor, was shown to be aberrantly expressed in pancreatic cancer by an unknown mechanism. It increased the angiogenesis of pancreatic cancer cells partially through transcriptional regulation of *PIM3*, a proto-oncogene with serine/threonine kinase activity [77,78]. PIM3 is constitutively expressed in pancreatic cancer, where it not only inhibits cell apoptosis by phosphorylating Bad [79] but also promotes pancreatic tumor neoangiogenesis [80].

### 3.6. Activating Invasion and Metastasis

Epithelial-mesenchymal transition (EMT) is a crucial way for tumor invasion and metastasis. Transcription factors *SNAIL*, *SLUG*, *TWIST* and *ZEB1/2* were identified in orchestrating EMT [81,82,83,84]. Furthermore, *miRNA-1271* has been found to have significantly decreased levels in pancreatic cancer tissues. It has been shown to increase EMT through inhibiting the expression of *TWIST* and *ZEB1* [85]. *EST-1* was also identified as a regulator of EMT in pancreatic cancer cells through *ZEB2*, thus increasing the motility of cancer cells [86,87].

### 3.7. Deregulating Cellular Energetics

Pancreatic cancer cells are deprived of oxygen and nutrients, which forces them to activate metabolic pathways to provide energy and maintain growth. Previous findings have confirmed that PDAC has an elevated glucose catabolism switch towards lactate production [88,89,90,91]. The mechanisms resulting in this are partially due to mutated *KRAS*. The *KRAS* gene regulates numerous enzymes involved in glutamine catabolism and glucose channeling towards glycolysis and Pentose Phosphate Pathway (PPP) (responsible for protein glycosylation and ribose production). *KRAS*^*G*12*D*^ mutation leads to mutations in its regulated metabolic enzymes. This results in activation of glucose and glutamine metabolisms through gain of activity of isocitrate dehydrogenase (IDH) and loss of activity of fumaratehydratase (FH) and succinate dehydrogenase(SDH) [92,93]. IDH, FH and SDH are major enzymes involved in the Krebs cycle.

### 3.8. Avoiding Immune Destruction

The immune system possesses the ability to regulate tumor progression whereby tumors are able to proliferate by escaping immune destruction. Significantly higher levels of hypoxia-inducible 1α (*HIF-1α*) have been identified in pancreatic carcinoma in comparison to chronic pancreatitis and normal pancreatic tissues. *HIF-1α* is inversely correlated with major histocompatibility complex class I chain-related (*MIC*) genes and is considered to be involved in tumor immune evasion [94]. Additionally, natural killer (NK) cells are important in non-specific immunoresponses in pancreatic cancer. Furthermore, elevated MMP9 expression in pancreatic cancer cells mediates natural killer cells dysfunction and facilitates immune evasion [95].

## 4. Effects of Pancreatic Cancer Genetics on Host Immunity

Genetic changes in pancreatic cancers have the ability to promote an immunosuppressive microenvironment to accelerate proliferation and progression of the tumor [96]. In this context, oncogenic *KRAS*, one of the major driver genes, is of particular importance. The oncogene drives immune privilege within the pancreatic tumor microenvironment, thus enabling pancreatic cancer progression through multiple mechanisms [97,98]. Of particular relevance to host anti-tumor immunity is the role of *KRAS* in upregulating granulocyte-macrophage colony-stimulating factor (*GM-CSF*). GM-CSF has been found at significantly high levels in many human PDAC cells and many studies have highlighted that oncogenic KRAS-driven GM-CSF expression in pancreatic intraepithelial neoplasia (PanIN) and invasive pancreatic cancer cells promote an influx of immunosuppressive myeloid cells, which inhibit adaptive immunity [99,100]. For example, Pylayeva-Gupta et al. recently reported that activation of mutated KRAS in pancreatic ductal cells promotes GM-CSF production. This leads to clonal expansion of immunosuppressive Gr1^+^ CD11b^+^ myeloid cells which are involved in cancer-associated inflammatory reactions. This in turn results in suppression of anti-tumor CD8^+^T cell-driven immunity [101]. Similarly, Bayne et al., demonstrated the equivalent correlation between GM-CSF and evasion of host anti-tumor T cell immunity through accumulation of Gr1^+^ CD11b^+^ myeloid cells in spontaneous murine models of PDAC [102]. Furthermore, McAllister et al., also indicated a role for mutant KRAS in promoting IL-17 production which was shown to be necessary for the development and progression of PanIN. The study demonstrated that mutant KRAS^G12D^ in vivo in the *Mist1*^*CreERT*2/+^; *LSL-KRAS*^*G*12*D*^ murine genetic model of PanIN drives expression of functional IL-17 receptors on PanIN epithelial cells. The oncogenic *KRAS* also induces infiltration of IL-17-producing immune cells, CD4^+^T and γδT within the pancreatic stroma, which stimulates PanIN initiation and development [103]. Overall, these findings implicate oncogenic *KRAS* and other genes that are stimulated through *KRAS* signaling pathways as promising targets for designing effective immunotherapies against pancreatic cancer.

In addition, Zhou et al. recently demonstrated that overexpression of human leucocyte antigen G (*HLA-G*) correlated with immune suppression and poor prognosis in pancreatic cancer patients. The study highlighted that HLA-G was found to be expressed at significantly higher levels in tumor tissues compared to normal tissues and this was associated with decreased levels of intratumoral CD3-positive tumor infiltrating lymphocytes [104]. The underlying mechanisms are not yet clarified.

## 5. Implications of Genomic Variations on Pancreatic Cancer Diagnosis and Treatment

The need for effective therapeutics for pancreatic cancer is extremely vital as this malignancy has one of the greatest rates of mortality in comparison to other types of cancer. In clinics, pancreatic cancer patients are treated with a stochastic approach based on the expertise and previous experiences of the clinician, as opposed to knowledge of cancer behavior and prognosis. This is one of the reasons underlying the poor prognosis of the disease. Consequently, this signifies the importance of understanding genomic variations in pancreatic cancer to enable clinicians to provide diagnostic and prognostic information to pancreatic cancer patients individually [89]. A ground breaking genomic analysis study on 24 distinct pancreatic cancers by Jones et al., uncovered an average of 63 genetic mutations per cancer, covering 12 different signal transduction pathways. The study emphasized the concept of the genetically heterogeneous nature of pancreatic cancer, which could in part account for its evident resistance to therapy and varying responses to treatment. As such, there is dire need for development of novel therapeutic strategies against specific genetic aberrations in order to enhance patient impact [105]. Of late, the use of genetic variants as biomarkers to guide targeted therapeutic approaches has been shown in varying types of cancer within the clinical setting. Conversely, clinical efforts steered by genetic variations have not yet been realized in pancreatic cancer. Moreover, it has been considered challenging to develop treatments to directly target the genetic abnormalities of the four major driver genes in pancreatic cancer: *KRAS*, *TP53*, *CDKN2A* and *SMAD4* due to a number of reasons such as multifunctionality and location within the cell [106]. Despite this, some promising results have been demonstrated, thus there are still ongoing efforts to develop novel gene-profile based therapeutic strategies in order to actualize precision medicine for pancreatic cancer [107].

In this part, we discuss the implications of genomic variations on pancreatic cancer diagnosis and treatment and recent advances in this field.

### 5.1. Diagnostic Biomarkers

The identification of biomarkers increases diagnostic accuracy and facilitates the classification of cancer subtypes. The carbohydrate antigen (CA 19-9) in serum is the most sensitive diagnostic marker of pancreatic cancer with 80% accuracy in clinics to date. Unfortunately, the principal limitation of this marker is that it has been shown to be elevated in patients with non-malignant obstructive jaundice and demonstrates low sensitivity for lesions less than 3 cm [108]. Thus, research into the discovery of new diagnostic markers is ongoing, especially for early diagnosis of pancreatic cancer. A review regarding the progress of early diagnosis for pancreatic cancer is published in this particular issue [109].

Telomerase activity has proven to be the most reliable diagnostic marker in pancreatic juice (PJ) samples as it can distinguish PDAC from chronic pancreatitis [75,110]. Moreover, Vascular endothelial growth factor (VEGF) A levels are also able to distinguish serous cystic neoplasms (SCN) from mucinous pancreatic cysts despite similar imaging results [111]. Mucinous pancreatic cysts, but not SCN, have the potential to progress to invasive pancreatic adenocarcinoma. Thus, levels of VEGFA, as a marker, could stratify patients that require surveillance or surgical interventions as well reduce expenses.

Through the study of genomic variations, distinct subtypes of pancreatic cancer have been defined. In particular, Bailey et al., recently analyzed the expression of 32 recurrently mutated genes in 456 PDAC patients and identified four subtypes: (1) squamous; (2) pancreatic progenitor; (3) immunogenic and (4) aberrantly differentiated endocrine exocrine (ADEX) [13]. Each subtype has varying molecular evolutions and responses to therapy. Similarly, Collissonet et al., also categorized PDAC into three subtypes based on molecular markers expressed in cancer cell lines: (1) classical, (2) quasi-mesenchymal (QM), and (3) exocrine-like. They also presented evidences of clinical outcome and therapeutic response differences between them [112]. QM-PDAC subtype lines were, on average, more sensitive to gemcitabine than classical subtypes while erlotinib was more effective in classical subtype cell lines. Hence, this evidence reinforces that classification of subtypes of pancreatic cancer can present clinicians with the avenue to provide personalized medicine approaches by stratifying patients for appropriate treatments based on their tumor subtype.

### 5.2. Prognostic Biomarkers

Prognostic biomarkers can aid clinicians in determining the risk of relapse and disease progression post-therapy. *KRAS*, *TP53*, *CDKN2A* and *SMAD4* are the four major driver genes in pancreatic cancer. Their status and the extent to which they coexist in an individual patients indicate disease progression and patient survival [113]. For example, Chen et al., demonstrated that survival of unresectable pancreatic cancer patients with *KRAS* mutations was poorer than patients with wild-type *KRAS* gene (3.9 months vs. 10.2 months, *p* < 0.001) [114]. Additionally, studies conducted by Xiang et al., showed that PDAC patients with mutant *TP*53^R172H^ and upregulated CAVIN1 represented the patient group with the shortest survival time after resection [115]. Furthermore, a recent study highlighted that PDAC patients with the expression of normal CDKN2A have better overall survival after curative resection [116]. Also, it has recently been shown that patients with loss of *SMAD4* have significantly poorer disease-free survival (mean 57.4 months vs. mean 17.6 months, *p =* 0.006) [117].

Moreover, other genes aside from the four major driver genes can also predict the postoperative survival of pancreatic cancer patients. For example, Kornmannet et al., established that patients with lower expression of *CCND1* had a median survival of 15.5 months compared with 6.5 months in patients with higher levels (*p* < 0.007) [118]. Furthermore, loss of 18q22.3/deletion of the *CPGL* gene has been elucidated as a poor prognostic marker in resected pancreatic cancer. Functional studies suggest that the *CPGL* gene is a growth suppressor gene in pancreatic cancer [119]. Additionally, SNPs of DNA mismatch repair (*MMR*) genes have the potential to predict patients’ clinical response to chemoradiotherapy and also can be prognostic markers for tumor respectability and resectable pancreatic cancer patients’ overall survival [120]. Moreover, Sausenet al., recently conducted large-scale genomic analyses of 24 PDAC tumors. The studies revealed that 20% of the patient samples displayed somatic mutations in chromatin-regulating genes, including *MLL*, *MLL2*, *MLL3* and *ARID1A*, and these mutations correlated with improved survival. Overall, these studies establish genetic predictors of prognosis for pancreatic cancer and provide new explorative avenues for therapeutic intervention.

### 5.3. Exploiting Genetic Variations to Improve Patient Responses to Gemcitabine

**Figure 2:**
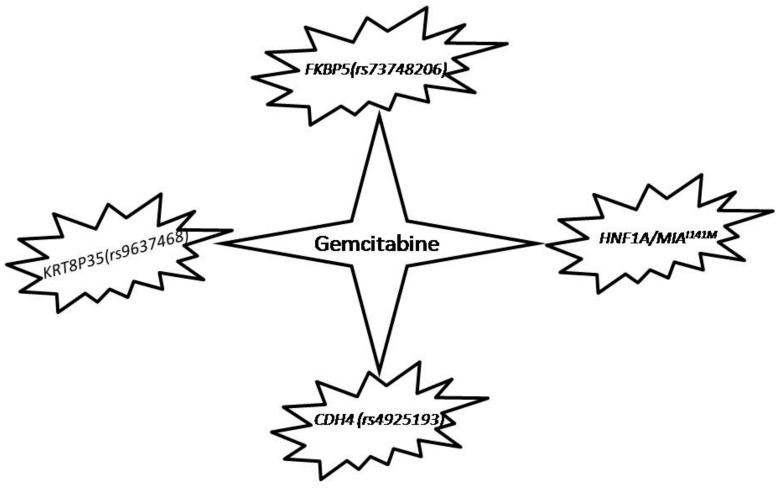
Genomic variations in pancreatic cancer that make patients more sensitive to Gemcitabine.

Currently, gemcitabine alone or in combination remains the first line therapy for advanced pancreatic cancer. However, response rates vary widely between patients, with many patients being insensitive to the drug [121]. This has paved the way for research into how genetic aberrations in pancreatic cancer impact on patient responses to gemcitabine. Traditionally, the emphasis in pharmaco genetic studies has been placed on genes in the gemcitabine metabolism pathways. These studies revealed that either expression of those genes or single nucleotide polymorphisms (SNPs) within them could only partially account for the observed variation in drug response [122]. Recently, Ellsworth et al., implicated FK506 binding proteins 5 (*FKBP5*), an immunophilin involved in protein folding, as a potential novel biomarker for predicting patient response rates to gemcitabine. The group conducted functional genomics studies which suggested that the rs73748206 SNP in *FKBP5*, may contribute to the varied patient responses to gemcitabine through upregulating *FKBP5* by greater binding to glucocorticoid receptor (GR), an established regulator of *FKBP5* expression. Similarly, through genotype-phenotype association analyses, Li et al., identified that four SNPs positioned in chromosomes 1, 3, 7 and 20, respectively, correlated with overall survival in patients who underwent treatment with gemcitabine. Further studies by the group delineated that two imputed SNPS, rs9637468 found in *KRT8P35* and rs4925193 in *CDH4* were associated with overall survival during gemcitabine therapy. In particular, three pancreatic cancer cells with *CDH4*-knockdown were significantly desensitized to treatment with gemcitabine, suggesting that *CDH4* may plays a role in differing responses to gemcitabine [123]. Moreover, recent genome-wide pleiotropy scan and transcriptome studies have established that the HNF1 homeobox A (*HNF1A*) gene has a key role in the progression of pancreatic cancer. Also, melanoma inhibitory activity 2 (*MIA2*) gene is one of the target genes of *HNF1A*. A recent study indicated that *HNF1A* and *MIA2* are expressed in a subset of human PDAC tissues and in vitro studies demonstrated that *HNF1A* induced *MIA2*. Of particular relevance is the group’s finding that PDAC cell lines that expressed *MIA2*^*I*141*M*^, a common germline variant of *MIA2*, had enhanced chemosensitivity to gemcitabine. This data could shed light on identifying subgroups of PDAC patients who may be more likely to benefit from gemcitabine treatment [124]. Overall, these data highlight novel biomarkers that may be used to predict responses to gemcitabine therapy (Figure 2), thus providing insight into strategies to stratify patients for treatment.

### 5.4. Therapeutic Inhibition of KRAS

Mutant *KRAS* plays a significant role in pancreatic cancer progression and in the majority of cases, oncogenic *KRAS* has the ability to initiate PDAC. Oncogenic *KRAS* is found exclusively in cancer cells with up to 90% of pancreatic cancer cells possessing the mutation [125]. Thus, research has been heavily invested in discovering treatments which target mutant *KRAS* in order to inhibit its effects (Table 2), however as yet, no KRAS inhibitors have progressed to usage in clinical settings. Directly inhibiting KRAS was initially considered to be an attractive approach for KRAS mutant PDAC, thus several efforts have been made to develop therapeutics to achieve this. Initially, farnesyl-transferase inhibitors (FTIs) were deemed to be the “wonder drug” for *KRAS*-driven PDAC. FTIs act by inhibiting farnesyl-transferase, which has the downstream effect of impeding KRAS activation, this is because KRAS is farnesylated, which enables it to interact with the membrane and become activated by RAS-guanine nucleotide exchange factors (GEFs). Despite an array of FTIs being investigated in clinics, such as Lonafarnib and Tipifarnib, the drugs have not demonstrated promising results. This could be attributed to variations among the three Ras proteins, as preclinical studies that generated enthusiasm for FTIs were conducted on HRAS-driven tumors. KRAS, as opposed to HRAS can seek alternative post-translational modifications for activation, such as being geranyl-geranylated once farnesyl-transferase is blocked. This provides KRAS with an alternate mechanism to enable association with the membrane and activation of its proteins [126]. Nevertheless, ongoing efforts in KRAS inhibition are still being pursued through other routes such as preventing KRAS from reaching cell membranes. In particular, *S*-trans, *trans*-FarnesylthiosalicylicAcid (FTS, salirasib) which inhibits RAS-dependent cell growth by dislodging all isoforms of RAS including mutant RAS, from the plasma membrane [29,127] has been investigated in several preclinical and phase II and III clinical trials and has shown some promise in pancreatic cancer. Another such small molecule inhibitor is Deltarasin, which acts by interacting with the farnesyl-binding pocket of PDEδ. PDEδ associates with farnesylated-KRAS and enables it to be translocated to the membrane. Hence, Deltarasin enables KRAS to be farnesylated but halts it from reaching the membrane. A recent study demonstrated that Deltarasin did indeed prevent KRAS from associating with the plasma membrane and decreased the proliferation of KRAS-driven PDAC cell lines [128].

In addition, mutations in KRAS hinder its ability to allow hydrolysis of GTP to GDP, hence forcing the protein to be locked in an active conformation. Thus, directly inhibiting the GTP-binding site of KRAS would be an ideal method to restrict KRAS signaling. However, small molecule inhibitors which have been investigated to date have not amounted to clinical success, thus KRAS is not generally considered to be druggable. This has driven efforts to investigate methods to indirectly target KRAS through downstream effectors in KRAS signaling pathways [125,129]. The MEK/MAPK and PI3K/AKT/mTOR pathways represent favorable pathways to be targeted therapeutically because they are the prevalent downstream pathways of KRAS and already have established clinical inhibitors available. Firstly, even though many MEK inhibitors, including CI-1040 and PD0325901 have been studied in clinical trials, they have been unsuccessful in delivering significant results. Moreover, drug targeting the PI3K/AKT/mTOR pathways, such as Everolimus, an inhibitor of mTOR, was able to impede tumor growth in vivo [130], however in a phase II study, it demonstrated little clinical success in PDAC patients resistant to gemcitabine. In contrast, LY294002, an inhibitor of PI3K was recently shown to promote apoptosis in vitro and prevent tumor proliferation in vivo. In addition, it was recently elucidated that cancers with activated *KRAS* and oncogenic *TP53* were unable to respond to mTOR inhibitors, conversely, tumors with *KRAS* activation and *PTEN* loss did respond to mTOR inhibitors [106]. Furthermore, Collissonet et al., recently reported that blocking MEK1/2 in orthotopically transplanted human and mouse PDAC cell lines potently prevented cancer cell growth. The study also highlighted that compensatory PI3K/AKT pathway activation occurred upon MEK1/2 inhibition, which can be justified by the known crosstalk between MEK and PI3K pathways in mutant KRAS tumors.This could be solved by a combinatory treatment of MEK1/2 with AKT inhibition, which has shown very impressive outcomes in preclinical studies in non-small cell lung cancer [129]. However, it is yet to be determined whether combining MAPK and PI3K pathway inhibition will provide synergistic effects in KRAS-driven endogenous PDAC in vivo [130].

### 5.5. Exploring Immunotherapy for Pancreatic Cancer Based on Genetic Variations

Immunotherapy has emerged as a highly promising and rapidly evolving strategy for cancer patient prognosis and has already shown clinical successes in melanoma and lung cancer patients. Despite this, immunotherapies for PDAC have not yet yielded much clinical benefit when administered as single agents. This low efficacy could be attributed to the highly fibrotic and immunosuppressive tumor microenvironment, which is prevalent in most human PDACs [131,132]. Nevertheless, the favorable clinical outcomes that the therapy poses have maintained considerable research efforts into immunotherapy for pancreatic cancer (Table 3).

Firstly, the identification of tumor antigens has resulted in the development of more specific and potent cancer vaccines. These vaccines are composed of the specific tumor antigen and are administered in order to augment the host’s natural immune defence against the antigen [133]. Of pancreatic cancer antigens, MUC1 has received the most interest due to its specific upregulation in pancreatic cancer cells and its correlation with tumor invasion and metastasis. However, clinical trials to date using MUC1-containing vaccines have not presented efficacious results, although the studies have shown an interesting association between MUC1 and immune biomarkers including CD38 (a marker for activated lymphocytes) and reduced T-regulatory cells (T-regs) levels. In addition, oncogenic *KRAS* has also received abundant attention in the field of cancer vaccines and was utilized as the first peptide vaccine to be studied in clinical trials targeting *KRAS* mutant pancreatic cancer. In similitude to MUC1-containing vaccines, vaccination approaches using mutant RAS peptides have not shown clinical benefits, however, more novel vaccination peptides are currently being analyzed clinically [98].

Furthermore, adoptive T-cell therapy represents another innovative immunotherapy for pancreatic cancer. This treatment involves expanding and activating of the patient’s T-cells Ex Vivo and then re-infusing them back into the patient. Adoptive T-cell transfer can be categorized into three groups depending on the source and method adopted for T-cell activation: (1) tumor infiltrating lymphocytes (TILs); (2) engineered T-cells which express a specific cancer T-cell receptor (TCR); and (3) T-cells which express a chimeric antigen receptor (CAR). Several targets have been studied in relation to adoptive T-cell therapy for pancreatic cancer; however the efficacy of this treatment is yet to be determined for these particular patients. One of these therapeutic targets includes MUC1 and a study that explored *MUC1*-specific cytotoxic T-lymphocytes in 20 patients demonstrated 19% 3 year-survival in those patients with resectable tumors, as well as elevated levels of effector lymphocytes and reduced T-regs [134]. Similarly, another molecule of interest is Mesothelin, a 40 kDa cell surface glycoprotein which is overexpressed in pancreatic cancer and is thought to be involved in metastasis. Initial data from human clinical trials have demonstrated that CAR T-cells specific for Mesothelin are well-tolerated and possess potential efficacy against pancreatic cancer [135].

Moreover, tumor-targeted oncolytic viruses (TOVs) have also appeared as a promising therapeutic for cancer immunotherapy, yet clinical successes have not been significant. The immunosuppressive tumor microenvironment might be a major factor for inhibiting TOV-induced anti-tumor immune effects. Principally, TOVs is designed to selectively eliminate cancer cells and produce systemic anti-tumor effects such as promoting long lasting anti-tumor immunity [5]. An example of this is Reolysin, a live replication-competent form of the reovirus serotype 3 Dearing strain. Reolysin has shown therapeutic efficacy in pancreatic cells with KRAS mutations, owing to its ability to replicate specifically in cells with activated KRAS ultimately resulting in lysis of KRAS-activated cancer cells [130].

Furthermore, monoclonal antibody (mAbs)-based immunotherapies have shown clinical efficacy in many cancers and have become a standard element of cancer therapeutics. MAbs elicit their anti-tumor effects through multiple methods, including direct targeting of the cancer cells; altering the host immune response; redirecting host immunity towards the cancerous cells; and delivering cytotoxic materials [136]. As previously mentioned, IL-17 plays a role in pancreatic cancer development often via immunosuppression, which has led to further studies to target and neutralize the cytokine for therapeutic benefit using mAbs [100]. Wu et al., recently elucidated that in pancreatic cancer patients, upregulation of IL-17 receptor B (*IL-17RB*) was highly associated with postoperative metastasis and negatively correlated with progression-free survival. Ex Vivo studies supported this discovery as it was demonstrated that IL-17RB and its ligand, IL-17 are vital elements for pancreatic cancer metastasis and progression. The study also highlighted a novel therapeutic approach for tackling pancreatic cancer through treatment with a newly derived monoclonal antibody against IL-17RB, which inhibited metastasis and improved survival in a mouse xenograft model [137,138]. Similarly, McAllister et al., revealed that monoclonal antibodies designed to neutralize IL-17 receptor A/IL-17 slowed the development of PanINs. These studies exemplify the importance of IL-17 inhibition as a targeted therapeutic approach for pancreatic cancer.

An abundance of recent research has given credence to the fact that the efficacy of immunotherapies is enhanced when deployed in combination. A recent study demonstrated that focal adhesion kinase 1 (FAK1) inhibitors strengthened the anti-tumor potency of adoptive T-cell transfer therapy and anti-programmed cell death protein 1 (PD-1) checkpoint inhibitors (in combination with gemcitabine). The study revealed that FAK1 activity was heightened in human PDAC cells and was associated with elevated levels of fibrosis and poor CD8^+^ cytotoxic T-cell infiltration, which have previously been identified as barriers to the success of immunotherapies. The group found that FAK inhibition using VS-4718 overcame the fibrotic and immunosuppressive PDAC tumor microenvironment, thus enabling the unresponsive KPC mouse model to be responsive to the immunotherapies [131]. Furthermore, another receptor implicated in immune modulation of pancreatic cancer is C-X-C chemokine receptor type 2 (CXCR2). Steele et al., recently determined that CXCR2 signaling is upregulated in neutrophils and myeloid-derived suppressor cells and this was associated with poor prognosis in PDAC. However, neutralization of CXCR2 via a clinically relevant CXCR2 small-molecule inhibitor prolonged tumor-free survival in mice models. Importantly, inhibition of CXCR2 enhanced sensitivity to anti-PD-1 therapy and this combination therapy improved T-cell infiltration and promoted survival [139,140].

Antibody therapy against immune-checkpoint, such as anti-CTLA-4, PD1 or PD-L1 antibodies, has achieved some impressive success in recent years, in particular in some patients with metastatic melanoma and lung cancer. This kind of therapy has demonstrated the possibility of relieving immune suppression in PDAC. We have recently summarized the progress and challenging in this field [5].

The clinical benefits of immunotherapy seen in melanoma and lung cancer warrant further studies within the pancreatic cancer field. Investigations are currently ongoing with the knowledge that combining immunotherapies with other immunotherapies such as immune checkpoint inhibitors as well as with chemotherapy may enhance treatment efficacy through synergistic effects [98].

## 6. Conclusions

Undoubtedly, the urgent need for novel and innovative therapeutics to tackle pancreatic cancer is of paramount importance in order to significantly improve prognosis of this almost universally lethal and devastating disease. The study of genomic variations is at the forefront of enhancing pancreatic cancer patient impact by providing an avenue for precision medicine. Understanding genomic variations in pancreatic cancer is crucial, as they are known to contribute to pancreatic carcinogenesis and provide fundamental knowledge for new and effective treatment strategies. Although it is well recognized that *KRAS*, *CDKN2A*, *SMAD4* and *TP53* are major driver genes in pancreatic cancer, thus far these genetic alterations have not been exploited effectively for therapeutic benefits. Hence, it is vital that further research is conducted into understanding how to target these major driver genes and their signaling pathways using immunotherapy and chemotherapy to enhance therapeutic benefits.

## Figures and Tables

**Table 1:** Summarization of genomic variation pathways that contribute to pancreatic carcinogenesis.

Pancreatic Tumor Traits	Genomic Variation Pathways
Sustained proliferation signaling	*PI3K/AKT*; *KRAS*; *PTEN*
Growth suppressor evasion	*TP53*; *RB*; *NF2/MERLIN*; *LKB1*
Cell death resistance	*CCND1*; *BCL2*; *TP53*; *BRCA2*; *miRNA216a/BECLIN1*
Enabling of replicative immortality	*PCDH10/hTERT*
Induction of angiogenesis	*ETS1/PIM3*
Activation of invasion and metastasis	*SNAIL*; *SLUG*; *miRNA-1271/TWIST*; *EST1/ZEB2*
Evasion of immune destruction	*HIF1α/MIC*; *MMP9*
Deregulated cellular energetics	*KRAS/IDH*; *FH*; *SDH*

**Table 2:** Mutant KRAS targeted drugs for pancreatic cancer treatment

Drugs	Mechanism	Efficacy
**FTIs (Lonafarnib and Tipifarnib)**	Inhibiting farnesylation of KRAS	Not promising
**FTS, salirasib**	Preventing KRAS from reaching cell membranes	Promising
**Deltarasin**	Enabling KRAS to be farnesylated but halting it from reaching the membrane	Decreasing the proliferation of KRAS-driven PDAC cell lines
**CI-1040 and PD0325901**	Inhibiting MEK/MAPK pathway downstream of KRAS	Not significant
**LY294002**	Inhibiting PI3K pathway downstream of KRAS	Promoting apoptosis in vitro and preventing tumor proliferation in vivo

**Table 3:** Immunotherapies for pancreatic cancer

Immunotherapies	Examples	Mechanism
**Tumor antigens identification**	MUC1; KRAS; Mesothelin, etc.	Development of more specific and potent cancer vaccines
**Adoptive T-cell therapy**	Tumor infiltrating lymphocytes (TILs)	Expanding and activating of the patient’s T-cells ex-vivo and then re-infusing them back into the patient to kill tumor cells
	Engineered T-cells which express a specific cancer T-cell receptor (TCR)	
	T-cells which express a chimeric antigen receptor (CAR)	
**Tumor-targeted oncolytic viruses (TOVs)**	Reolysin, etc.	Selectively eliminating cancer cells and producing systemic anti-tumor effects such as promoting long lasting anti-tumor immunity
**Monoclonal antibody**	IL17RB; IL17RA, etc.	Direct targeting of the cancer cells; altering the host immune response; redirecting host immunity towards the cancerous cells; and delivering cytotoxic materials
**Immune checkpoint therapy**	CTLA4; PD1, etc.	Enhancing T cells function
